# Supplementary material for: Estimating the prevalence and risk of COVID-19 among international travelers and evacuees of Wuhan through modeling and case reports
Source: PLoS One. 2020 Jun 23;15(6):e0234955. doi: 10.1371/journal.pone.0234955 (PMC7310725; doi:10.1371/journal.pone.0234955)
Supplement: S1 File — (PDF) [file pone.0234955.s002.pdf]

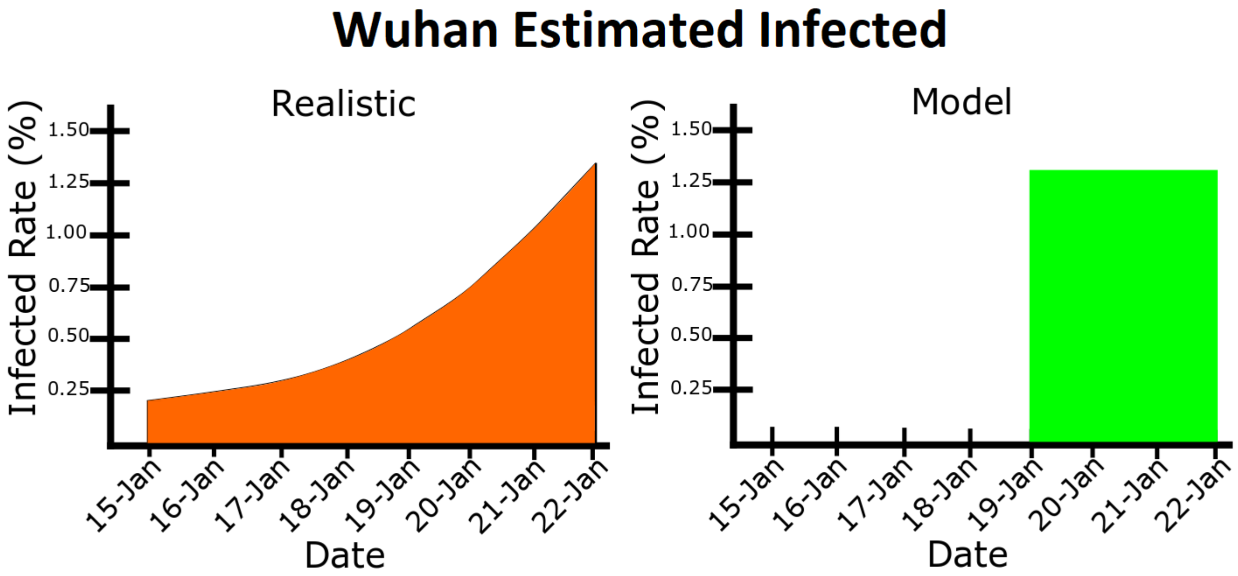

Fig S1. Realistic vs Model of Wuhan Estimated Infection. To approximate the infected rate, we used the model scenario in our calculations to simplify our estimation of the infected rate. The realistic scenario is only a representation and not a precise depiction of the COVID-19 infected rate in Wuhan.

Country	Patient Number	Flight Date	Confirmation Date	References
Japan	1	1/6/2020	1/15/2020	1
Japan	2	1/19/2020	1/24/2020	2
Japan	3	1/18/2020	1/25/2020	3
Japan	4	1/22/2020	1/26/2020	4
Japan	5	1/20/2020	1/28/2020	5
Japan	7	1/21/2020	1/28/2020	6
Japan	10	1/13/2020	1/30/2020	7
Japan	11	1/20/2020	1/30/2020	8
Japan	12	1/22/2020	1/30/2020	9
Japan	17	1/21/2020	2/4/2020	10
Japan	19	1/22/2020	2/4/2020	11
Japan	20	1/21/2020	2/5/2020	12
Singapore	1	1/20/2020	1/23/2020	13
Singapore	2	1/21/2020	1/24/2020	14
Singapore	3	1/20/2020	1/24/2020	14
Singapore	4	1/22/2020	1/25/2020	15
Singapore	5	1/18/2020	1/27/2020	16
Singapore	6	1/19/2020	1/28/2020	17
Singapore	7	1/23/2020	1/28/2020	17
Singapore	8	1/19/2020	1/29/2020	18
Singapore	9	1/19/2020	1/29/2020	18
Singapore	10	1/20/2020	1/29/2020	18
Singapore	11	1/22/2020	1/29/2020	19
Singapore	12	1/22/2020	1/29/2020	19
Singapore	13	1/21/2020	1/30/2020	19
Singapore	14	1/26/2020	1/30/2020	20
Singapore	16	1/22/2020	1/31/2020	20
Singapore	18	1/22/2020	2/1/2020	21
Singapore	26	1/21/2020	2/4/2020	22
Korea	1	1/19/2020	1/20/2020	23
Korea	2	1/22/2020	1/24/2020	24
Korea	3	1/20/2020	1/26/2020	25
Korea	4	1/20/2020	1/27/2020	26
Korea	5	1/24/2020	1/30/2020	27
Korea	7	1/23/2020	1/31/2020	28
Korea	8	1/23/2020	1/31/2020	28
Korea	15	1/20/2020	2/2/2020	29
Korea	23	1/23/2020	2/6/2020	30
Korea	26	1/31/2020	2/9/2020	31,32
Korea	27	1/31/2020	2/9/2020	31,32

Table S2. Exported Cases from China. We looked at the flight dates of infected travelers from China to Japan, Singapore, and Korea before February 14th, 2020. These travelers do not include evacuees.

Country	Evacuees	Infected	1st Evacuation Date	Infected Rate	References
Singapore	266	6	1/30/2020	2.26%	33
Malaysia	107	2	2/4/2020	1.87%	34
Italy	56	1	2/3/2020	1.79%	35
Germany	124	2	1/31/2020	1.61%	36
Japan	763	9	1/29/2020	1.18%	37
UK	199	1	1/31/2020	0.50%	38
US	800	3	1/29/2020	0.38%	39
Korea	701	1	1/30/2020	0.14%	40
Australia	500	0	2/4/2020	0.00%	41
Vietnam	30	0	2/10/2020	0.00%	42
Thailand	138	0	2/1/2020	0.00%	43
India	647	0	2/1/2020	0.00%	44
Philippines	30	0	2/1/2020	0.00%	45
Canada	398	0	2/6/2020	0.00%	46
France	302	0	1/31/2020	0.00%	47
Russia	140	0	2/5/2020	0.00%	48

Table S3. Evacuees from Wuhan. We compiled the number of evacuees and the number of infected evacuees from each country.

Flight Date	Number of Evacuees	Infected	Infected Rate	References
1/29/2020	206	4	1.9%	37,49
1/30/2020	210	2	1.0%	37,50
1/31/2020	149	2	1.3%	37,51
2/7/2020	198	1	0.5%	37,52
Total	763	9	1.2%	37

Table S4. Japan's Evacuations. The first 4 evacuations to Japan were recorded with the number of evacuees and infected evacuees. Infected evacuee tested positive by RT-PCR for SARS-COV-2.

Flight Date	Number of Passengers	Infected	Infected Rate	References
1/30/2020	92	5	5.4%	53-56
2/9/2020	174	1	0.6%	57

Table S5. Singapore Evacuations. The first 2 evacuations to Singapore were recorded with the number of evacuees and infected evacuees. Infected evacuee tested positive by RT-PCR for SARS-COV-2.

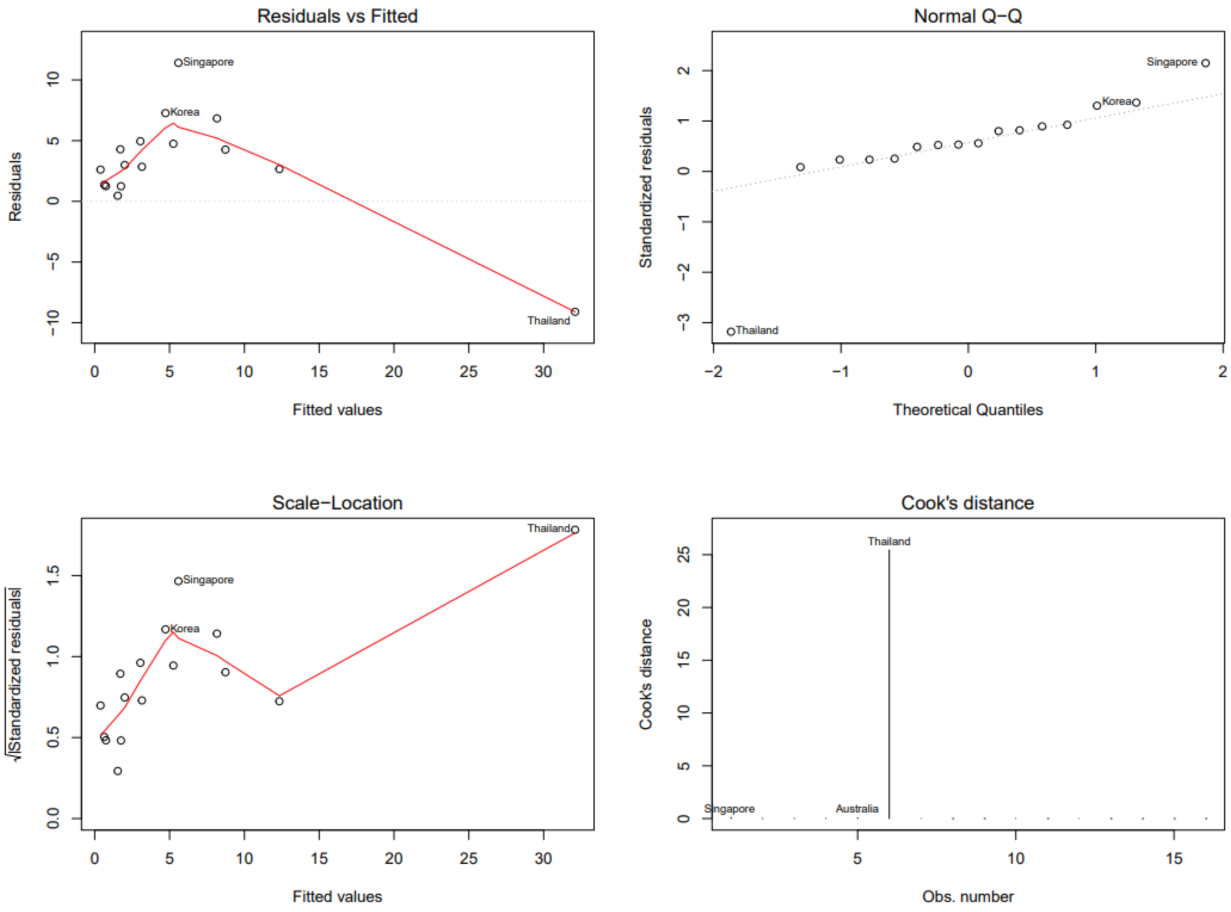

Fig S2. Linear Regression Plots of Infected Traveler Model Including Thailand. To test whether our model of infected travelers fit the four assumptions of linear regression model, we generated diagnostic plots to examine the fit of the line. Thailand appeared to be a significant outlier that we eventually chose to exclude because it appears to be discordant compared to the other points.

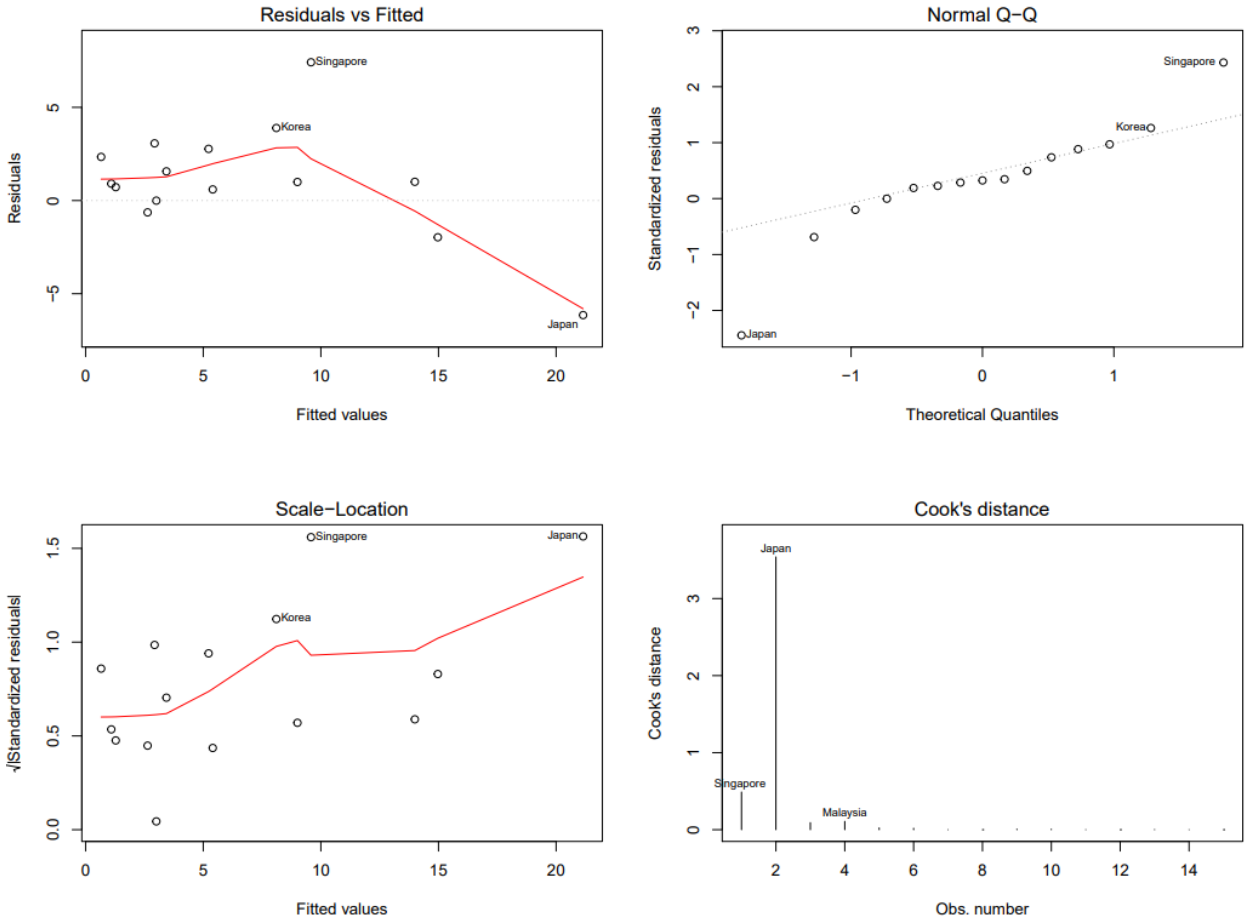

Fig S3. Linear Regression Plots of Infected Traveler Model Excluding Thailand. We also generated diagnostic plots to examine the fit of the linear regression without Thailand. We found that the fit was much better although Japan and Singapore appear to still deviate from the line significantly more than other countries. However, we believe data from both countries are valuable and should be included in the model. We believe a linear model is still appropriate after the diagnostic plots as the assumptions of linear regression have not been significantly violated.

References

1. Outbreak of patients with pneumonia associated with new coronavirus (1st case): Japan Ministry of Health; 2020. Available from: https://www.mhlw.go.jp/stf/newpage_08906.html.
2. Outbreak of patients with pneumonia associated with new coronavirus (2nd case): Japan Ministry of Health; 2020. Available from: https://www.mhlw.go.jp/stf/newpage_09079.html.
3. Outbreak of patients with pneumonia associated with new coronavirus (3rd' case): Japan Ministry of Health; 2020. Available from: https://www.mhlw.go.jp/stf/newpage_09099.html
4. Outbreak of patients with pneumonia associated with new coronavirus (4th case): Japan Ministry of Health; 2020. Available from: https://www.mhlw.go.jp/stf/newpage_09100.html
5. Outbreak of patients with pneumonia associated with new coronavirus (5th case): Japan Ministry of Health; 2020. Available from: https://www.mhlw.go.jp/stf/newpage_09154.html
6. Outbreak of patients with pneumonia associated with new coronavirus (7th case): Japan Ministry of Health; 2020. Available from: https://www.mhlw.go.jp/stf/newpage_09158.html
7. Outbreak of patients with pneumonia associated with new coronavirus (10th case): Japan Ministry of Health; 2020. Available from: https://www.mhlw.go.jp/stf/newpage_09236.html
8. Outbreak of patients with pneumonia associated with new coronavirus (11 case): Japan Ministry of Health; 2020. Available from: https://www.mhlw.go.jp/stf/newpage_09237.html
9. Outbreak of patients with pneumonia associated with new coronavirus (12th case): Japan Ministry of Health; 2020. Available from: https://www.mhlw.go.jp/stf/newpage_09239.html
10. Outbreak of patients with pneumonia associated with new coronavirus (17th case): Japan Ministry of Health; 2020. Available from: https://www.mhlw.go.jp/stf/newpage_09310.html
11. Outbreak of patients with pneumonia associated with new coronavirus (19th case): Japan Ministry of Health; 2020. Available from: https://www.mhlw.go.jp/stf/newpage_09312.html
12. Outbreak of patients with pneumonia associated with new coronavirus (20th case): Japan Ministry of Health; 2020. Available from: https://www.mhlw.go.jp/stf/newpage_09333.html
13. CONFIRMED IMPORTED CASE OF NOVEL CORONAVIRUS INFECTION IN SINGAPORE; MULTI-MINISTRY TASKFORCE RAMPS UP PRECAUTIONARY MEASURES: Singapore Ministry of Health; 2020. Available from: https://www.moh.gov.sg/news-highlights/details/confirmed-imported-case-of-novel-

coronavirus-infection-in-singapore-multi-ministry-taskforce-ramps-up-precautionary-measures.
14. TWO MORE CASES OF CONFIRMED IMPORTED CASE OF NOVEL CORONAVIRUS INFECTION IN SINGAPORE: Singapore Ministry of Health; 2020. Available from: https://www.moh.gov.sg/news-highlights/details/two-more-cases-of-confirmed-imported-case-of-novel-coronavirus-infection-in-singapore
15. FOURTH CONFIRMED IMPORTED CASE OF WUHAN CORONAVIRUS INFECTION IN SINGAPORE: Singapore Ministry of Health; 2020. Available from: https://www.moh.gov.sg/news-highlights/details/fourth-confirmed-imported-case-of-wuhan-coronavirus-infection-in-singapore
16. FIFTH CONFIRMED IMPORTED CASE OF WUHAN CORONAVIRUS INFECTION IN SINGAPORE: Singapore Ministry of Health; 2020. Available from: https://www.moh.gov.sg/news-highlights/details/fifth-confirmed-imported-case-of-wuhan-coronavirus-infection-in-singapore-27Jan
17. TWO MORE CONFIRMED IMPORTED CASES OF WUHAN CORONAVIRUS INFECTION IN SINGAPORE; 2020. Available from: https://www.moh.gov.sg/news-highlights/details/two-more-confirmed-imported-cases-of-wuhan-coronavirus-infection-in-singapore
18. THREE MORE CONFIRMED IMPORTED CASES OF WUHAN CORONAVIRUS INFECTION IN SINGAPORE: Singapore Ministry of Health; 2020. Available from: https://www.moh.gov.sg/news-highlights/details/three-more-confirmed-imported-cases-of-wuhan-coronavirus-infection-in-singapore
19. THREE MORE CONFIRMED IMPORTED CASES OF WUHAN CORONAVIRUS INFECTION IN SINGAPORE: Singapore Ministry of Health; 2020. Available from: https://www.moh.gov.sg/news-highlights/details/three-more-confirmed-imported-cases-of-wuhan-coronavirus-infection-in-singapore-30Jan
20. THREE MORE CONFIRMED IMPORTED CASES OF WUHAN CORONAVIRUS INFECTION IN SINGAPORE: Singapore Ministry of Health; 2020. Available from: https://www.moh.gov.sg/news-highlights/details/three-more-confirmed-imported-cases-of-wuhan-coronavirus-infection-in-singapore-31-jan
21. TWO MORE CONFIRMED IMPORTED CASES OF NOVEL CORONAVIRUS INFECTION IN SINGAPORE: Singapore Ministry of Health; 2020. Available from: https://www.moh.gov.sg/news-highlights/details/two-more-confirmed-imported-cases-of-novel-coronavirus-infection-in-singapore
22. FOUR MORE CONFIRMED CASES OF NOVEL CORONAVIRUS INFECTION IN SINGAPORE: Singapore Ministry of Health; 2020. Available from: https://www.moh.gov.sg/news-highlights/details/four-more-confirmed-cases-of-novel-coronavirus-infection-in-singapore
23. Confirmation of confirmed cases of new influx of new coronavirus in the quarantine phase Raise and respond to the infectious disease crisis alert to the 'attention' level: Korean Ministry of Health and Welfare; 2020. Available from: http://www.mohw.go.kr/react/al/sal0301vw.jsp?PAR_MENU_ID=04&MENU_ID=0403&page=39&CONT_SEQ=352435

24. 24. New coronavirus confirmed as the second domestic patient: Korean Ministry of Health and Welfare; 2020. Available from: http://www.mohw.go.kr/react/al/sal0301vw.jsp?PAR_MENU_ID=04&MENU_ID=0403&page=37&CONT_SEQ=352506
25. New coronavirus confirmed as the third patient in Korea: Korean Ministry of Health and Welfare; 2020. Available from: http://www.mohw.go.kr/react/al/sal0301vw.jsp?PAR_MENU_ID=04&MENU_ID=0403&page=37&CONT_SEQ=352511
26. Current Status of New Coronaviruses in Korea: Korean Ministry of Health and Welfare; 2020. Available from: http://www.mohw.go.kr/react/al/sal0301vw.jsp?PAR_MENU_ID=04&MENU_ID=0403&page=37&CONT_SEQ=352516
27. Confirmation of 2 additional patients with new coronavirus: Korean Ministry of Health and Welfare; 2020. Available from: http://www.mohw.go.kr/react/al/sal0301vw.jsp?PAR_MENU_ID=04&MENU_ID=0403&page=36&CONT_SEQ=352575
28. New Coronavirus Domestic Outbreak Status Notice (January 31): Korean Ministry of Health and Welfare; 2020. Available from: http://www.mohw.go.kr/react/al/sal0301vw.jsp?PAR_MENU_ID=04&MENU_ID=0403&page=36&CONT_SEQ=352609
29. Domestic occurrence of new coronavirus: Korean Ministry of Health and Welfare; 2020. Available from: http://www.mohw.go.kr/react/al/sal0301vw.jsp?PAR_MENU_ID=04&MENU_ID=0403&page=35&CONT_SEQ=352642
30. Current status of new coronavirus in Korea (as of February 6): Korean Ministry of Health and Welfare; 2020. Available from: http://www.mohw.go.kr/react/al/sal0301vw.jsp?PAR_MENU_ID=04&MENU_ID=0403&page=37&CONT_SEQ=352511
31. Domestic occurrence of new coronavirus infection (as of February 9): Korean Ministry of Health and Welfare; 2020. Available from: http://www.mohw.go.kr/react/al/sal0301vw.jsp?PAR_MENU_ID=04&MENU_ID=0403&page=33&CONT_SEQ=352786
32. Domestic occurrence of new coronavirus infection (as of February 9): Korean Ministry of Health and Welfare; 2020. Available from: http://www.mohw.go.kr/react/al/sal0301vw.jsp?PAR_MENU_ID=04&MENU_ID=0403&page=33&CONT_SEQ=352783
33. Aravindan A. Singapore evacuating 174 on second flight from Wuhan: Reuter; 2020. Available from: https://www.reuters.com/article/us-china-health-singapore/singapore-evacuating-174-on-second-flight-from-wuhan-idUSKBN2020EY.
34. Four COVID-19 patients discharged in Malaysia, all evacuees from Wuhan complete quarantine: CNA; 2020. Available from: https://www.channelnewsasia.com/news/asia/malaysia-covid-19-recover-evacuees-wuhan-complete-quarantine-12446820.

35. Italian evacuee from Wuhan positive for novel coronavirus: Inquirer; 2020. Available from: https://newsinfo.inquirer.net/1225850/italian-evacuee-from-wuhan-positive-for-novel-coronavirus.
36. Coronavirus: German evacuation flight from China carried two infected people: dw.com; 2020. Available from: https://www.dw.com/en/coronavirus-german-evacuation-flight-from-china-carried-two-infected-people/a-52229955.
37. About the present situation of new coronavirus infectious disease and correspondence of Ministry of Health, Labor and Welfare (February 12, 2012 version): Japan Ministry of Health; 2020. Available from: https://www.mhlw.go.jp/stf/newpage_09450.html.
38. Coronavirus: Fourth patient in UK diagnosed: BBC; 2020. Available from: https://www.bbc.com/news/uk-51430654.
39. Feuer W. CDC confirms 15th US coronavirus case, a Wuhan evacuee quarantined at Texas military base: CNBC; 2020. Available from: https://www.cnbc.com/2020/02/13/cdc-confirms-15th-us-case-of-coronavirus-among-wuhan-evacuees-under-quarantine-at-texas-military-base.html.
40. Chan-kyong P. Coronavirus: South Korean returnee from Wuhan tests positive, Malaysia confirms one more: SCMP; 2020. Available from: https://www.scmp.com/news/asia/east-asia/article/3049516/coronavirus-south-korean-returnee-wuhan-tests-positive-countrys.
41. Xiao B, Choahan N. Australians in Wuhan told no more evacuation flights planned out of coronavirus epicentre abc.au2020. Available from: https://www.abc.net.au/news/2020-02-12/australian-citizens-wuhan-no-planned-evacuations-coronavirus/11955520.
42. Plane brings 30 Vietnamese evacuees from Wuhan: Saigon Times; 2020. Available from: https://english.thesaigontimes.vn/74601/plane-brings-30-vietnamese-evacuees-from-wuhan.html.
43. Thepgumpanat P, Tanakasempipat P. Thailand evacuates 138 from virus-hit Wuhan: Reuters; 2020. Available from: https://www.reuters.com/article/us-china-health-thailand/thailand-evacuates-138-from-virus-hit-wuhan-idUSKBN1ZY0ZF.
44. Indian envoy describes evacuation of Indians from virus-hit Wuhan a logistical nightmare: economictimes.indiatimes; 2020. Available from: https://economictimes.indiatimes.com/news/international/world-news/indian-envoy-describes-evacuation-of-indians-from-virus-hit-wuhan-a-logistical-nightmare/articleshow/74067472.cms.
45. Philippines evacuates 30, including infant, from virus-hit Wuhan: Reuters; 2020. Available from: https://www.reuters.com/article/us-china-health-philippines/philippines-evacuates-30-including-infant-from-virus-hit-wuhan-idUSKBN20304F.
46. Jackson H. 2nd evacuation flight chartered by Ottawa leaves Wuhan with 185 on board: Global News; 2020. Available from: https://globalnews.ca/news/6530909/coronavirus-quarantine-flight-crew-released/.
47. 36 evacuated from China to France show virus symptoms: minister: France24; 2020. Available from: https://www.france24.com/en/20200203-36-evacuated-from-china-to-france-show-virus-symptoms-minister.

48. Russia's strangest reality show: Siberian quarantine videos shared on Instagram: The Guardian; 2020. Available from: https://www.theguardian.com/world/2020/feb/06/we-dont-leave-our-rooms-russians-quarantined-in-siberia-take-to-instagram.
49. About outbreak of subclinical pathogen carrier (*) related to new coronavirus: Japan Ministry of Health; 2020. Available from: https://www.mhlw.go.jp/stf/newpage_09273.html
50. About the test results of returning Japanese people on charter flights related to the new coronavirus: Japan Ministry of Health; 2020. Available from: https://www.mhlw.go.jp/stf/newpage_09202.html
51. Outbreaks of new coronavirus-related patients (14th and 15th cases) and asymptomatic pathogen carriers (*): Japan Ministry of Health; 2020. Available from: https://www.mhlw.go.jp/stf/newpage_09278.html
52. Status of Japanese returnees on charter flights related to the new coronavirus: Japan Ministry of Health; 2020. Available from: https://www.mhlw.go.jp/stf/newpage_09395.html
53. THREE MORE CONFIRMED IMPORTED CASES OF WUHAN CORONAVIRUS INFECTION IN SINGAPORE: Singapore Ministry of Health; 2020. Available from: https://www.moh.gov.sg/news-highlights/details/three-more-confirmed-imported-cases-of-wuhan-coronavirus-infection-in-singapore-31-jan
54. TWO MORE CONFIRMED IMPORTED CASES OF NOVEL CORONAVIRUS INFECTION IN SINGAPORE: Singapore Ministry of Health; 2020. Available from: https://www.moh.gov.sg/news-highlights/details/two-more-confirmed-imported-cases-of-novel-coronavirus-infection-in-singapore
55. FOUR MORE CONFIRMED CASES OF NOVEL CORONAVIRUS INFECTION IN SINGAPORE: Singapore Ministry of Health; 2020. Available from: https://www.moh.gov.sg/news-highlights/details/four-more-confirmed-cases-of-novel-coronavirus-infection-in-singapore
56. ONE MORE CASE DISCHARGED; TWO NEW CASES OF NOVEL CORONAVIRUS INFECTION CONFIRMED: Singapore Ministry of Health; 2020. Available from: https://www.moh.gov.sg/news-highlights/details/one-more-case-discharged-two-new-cases-of-novel-coronavirus-infection-confirmed-10feb
57. FIVE MORE CASES DISCHARGED; TWO NEW CASES OF COVID-19 INFECTION CONFIRMED: Singapore Ministry of Health; 2020. Available from: https://www.moh.gov.sg/news-highlights/details/five-more-cases-discharged-two-new-cases-of-covid-19-infection-confirmed
