# Supplementary material for: Estimating the prevalence and risk of COVID-19 among international travelers and evacuees of Wuhan through modeling and case reports
Source: PLoS One. 2020 Jun 23;15(6):e0234955. doi: 10.1371/journal.pone.0234955 (PMC7310725; doi:10.1371/journal.pone.0234955)
Supplement: Attachment — Submitted filename: Response to Revision Request Final.pdf [file pone.0234955.s004.pdf]

Response to Revision Requests

Editors Comments

1. Add a conclusions section to the manuscript where you summarize your findings and include both policy recommendations and future lines of academic research. My earlier comment #5 has not been addressed fully. You have included policy recommendations in your last paragraph but not what/how should researchers continue investigating. In light of your findings, what should academics pursue or extend from your work? My suggestion was to use R2#11 related to modeling. Your model is a linear regression, which has known limitations (omitted variable bias, heteroscedasticity, unobserved heterogeneity). Please acknowledge this limitation and offer some suggestions to other academics.

We appreciate the suggestion, and we have expanded our manuscript to include a conclusions section with our findings and recommendations (lines 292-304). Additionally, we talk about what researchers and government can do to collaborate for future research (lines 272-291). We acknowledge that our model has limitations (lines 206-213), some of which are explicit (lines 204-206), and that researchers can adapt or develop other models (lines 285-288) depending on data availability. Additionally, we used diagnostic plots to evaluate the assumptions of the linear regression (normality, homoscedasticity, independence, and linearity) which is included in our figures S2 and S3.

2. Provide a roadmap of the paper as the last paragraph of the introduction (that includes the new conclusions section).

We agree with the comment and have made our last paragraph of the introduction the roadmap of our paper (lines 70-80).
